# Awareness of and Satisfaction with Governmental COVID-19 Support Among Foreign Residents in Japan: A Cross-Sectional Study

**DOI:** 10.3390/healthcare14101279

**Published:** 2026-05-08

**Authors:** Shizuko Arima, Rie Ogasawara, Daisuke Onozuka

**Affiliations:** 1 Faculty of Nursing and Rehabilitation, Konan Women’s University, Kobe 658-0001, Hyogo, Japan; 2 Department of Global and Innovative Medicine, Graduate School of Medicine, The University of Osaka, Suita 565-0871, Osaka, Japan; rie.ogasawara@gim.med.osaka-u.ac.jp; 3 Department of Post-Infectious Diseases Therapeutics, Graduate School of Medicine, The University of Osaka, Suita 565-0871, Osaka, Japan; onozukad@hp-infect.med.osaka-u.ac.jp

**Keywords:** COVID-19, foreign residents, government support, service awareness, satisfaction, length of residence, Japan

## Abstract

**Highlights:**

**What are the main findings?**

- Awareness of government support was the strongest predictor of satisfaction among foreign residents.
- Satisfaction was high even among non-users who were aware of support systems.

**What is the implication of the main finding?**

- Length of residence influenced satisfaction patterns.
- Tailored strategies are needed to improve access to government support.

**Abstract:**

**Background**: Foreign residents in Japan faced various barriers that hindered their access to governmental COVID-19 (Coronavirus Disease 2019) support, which may have influenced their satisfaction with available services and overall well-being. However, limited evidence exists on how awareness of such support relates to satisfaction. This study examined the association between awareness of governmental COVID-19 support and satisfaction among foreign residents living in Japan. **Methods**: A cross-sectional online survey was conducted between September and November 2023 using a commercial online survey panel of foreign residents in Japan. Satisfaction with governmental support was assessed using a 10-point scale and dichotomized at the median. Signal detection analysis was applied to identify factors associated with higher satisfaction. **Results**: Among 427 respondents, 400 (93.7%) reported receiving COVID-19 vaccination. Satisfaction with government support was assessed using a 10-point scale, with a mean score of 5.8. Awareness of support was the strongest predictor of satisfaction, and length of residence was an additional determinant among those aware of support. Distinct demographic and socioeconomic patterns were observed across awareness and residence groups. **Conclusions**: Awareness of governmental COVID-19 support played a key role in shaping satisfaction among foreign residents, regardless of service use. Tailored, group-specific approaches considering length of residence and individual characteristics may improve access to support and enhance well-being.

## 1. Introduction

Japan is experiencing rapid population aging and a declining birth rate, which have resulted in a severe labor shortage. In response to this demographic challenge, the Japanese government has expanded the acceptance of foreign nationals.

In April 2019, the revised Immigration Control and Refugee Recognition Act came into effect, enabling the large-scale acceptance of foreign workers in addition to technical intern trainees and international students. Before and during the coronavirus disruptions, this demographic shift was already evident. The foreign resident population reached approximately 2.93 million in 2019 [1]. Among them, in 2019, Chinese nationals accounted for approximately 28%, followed by Korean nationals (17%) and Vietnamese nationals (13%). In terms of residence status, permanent residents and similar statuses constituted the largest group (52%), followed by technical intern trainees (27%), international students (25%), those with “Engineer/Specialist in Humanities/International Services” status (19%), dependents (14%), and so on. During the COVID-19 pandemic, however, strict border control measures and travel restrictions significantly affected international migration to Japan. As a result, the number of foreign residents temporarily declined during 2020 and 2021 after several consecutive years of increase, decreasing to about 2.76 million by the end of 2021. This decline reflected the immediate impact of entry restrictions and reduced international mobility during the pandemic.

The rapid increase in the foreign resident population has also generated new challenges related to access to government support. Some foreign residents face difficulties in accessing essential public services, leading to delayed medical consultations and unmet health needs. These challenges became particularly evident during the COVID-19 pandemic, when infection clusters occurred in dormitories and factories where foreign workers lived in group settings, highlighting the vulnerability of health and living conditions within foreign resident communities.

A systematic review identified several key facilitators, including accessible information provision, affordable language interpretation services, culturally appropriate healthcare, and the presence of social networks [2]. Nevertheless, disparities in access to government support persist. While information provision is essential for reducing these disparities, differences in age, Japanese language proficiency, internet access, and the methods used by administrative agencies to disseminate information contribute to unequal levels of information reception and comprehension. Mastering the Japanese language poses a substantial challenge for many foreign residents, representing a major barrier to accessing support information. Although local municipalities and volunteer groups across Japan offer community-based Japanese language courses to support integration, access to these opportunities is often limited by scheduling conflicts with work commitments, geographical constraints, and a shortage of certified language instructors. Consequently, the significant time and effort required to achieve language proficiency leave many foreign residents struggling to independently comprehend complex administrative procedures and public health announcements, thereby heightening their need for multilingual support [2,3].

To address these challenges, researchers and practitioners have explored practical approaches that leverage community networks and foreign resident support personnel. Foreign community organizations and local networks play an important role in disseminating government support information. These networks help mitigate barriers related to language and digital access. In addition, foreign peer supporters and navigators assist in providing information and guiding residents through administrative procedures. Ikeda [4] reported cases in which navigators supported foreign residents in obtaining COVID-19 vaccination vouchers, highlighting the importance of coordinated administrative support. Furthermore, case studies of foreign resident support during the COVID-19 pandemic demonstrated that collaboration among public health centers, medical institutions, and foreign resident consultation centers enabled access to PCR testing [5].

During the COVID-19 pandemic, the Japanese government and local municipalities implemented various public support measures to mitigate the social and health impacts of the pandemic. These measures included financial assistance programs such as the Special Cash Payments, livelihood support, and public health services [6,7]. Vaccination programs were widely implemented as a major healthcare-related public support measure to prevent severe illness and reduce the spread of infection. COVID-19 vaccines were provided free of charge to residents, including foreign residents, and local governments played an important role in disseminating information and facilitating access to vaccination services through multilingual communication and community outreach.

Although knowledge has accumulated regarding the relationship between community network involvement and access to government support among foreign residents, gaps remain. Differences in satisfaction with government support and the factors underlying these differences—such as individual attributes and living environments—have not been fully elucidated. Therefore, this study focuses on awareness of government support as a key determinant of satisfaction among foreign residents. The study aims to examine the associations between service awareness, length of residence, and social attributes. By doing so, this research seeks to identify effective strategies for information dissemination and support for populations with limited access to governmental support, ultimately contributing to the improvement of well-being among foreign residents.

## 2. Materials and Methods

### 2.1. Definition of Terms

In this study, the term foreign residents refers to non-Japanese nationals categorized as “mid- to longer-term residents” and “special permanent residents” in the statistics published by the Immigration Services Agency of Japan [1]. In these statistics, “mid- to longer-term residents” exclude, for example, persons granted a period of stay of three months or less, those with “Temporary Visitor” status, and those with “Diplomat” or “Official” status [1].

In Japan, foreign residents who legally stay for more than three months and have an address in a municipality are required to complete resident registration and are recorded in the municipal Basic Resident Register (i.e., a resident record, jūminhyō, is created). As registered residents, they are subject to local taxation (e.g., individual inhabitant tax) and are covered by social security programs, including public health insurance. Foreign residents who are not covered by employment-based health insurance are generally required to enroll in the municipal National Health Insurance.

### 2.2. Survey Method

Data were collected between September and November 2023. Participants were recruited using the “foreign residents in Japan” panel managed by a private research company (Asmark Inc.). Eligible participants were defined as foreign nationals aged 20 years or older who were registered as mid- to longer-term residents or special permanent residents in Japan. Individuals staying for less than three months (e.g., temporary visitors) were explicitly excluded at the recruitment stage. The online survey system was configured to prevent duplicate entries from the same user. Eligible participants received an invitation to participate in the study through the panel system. Researchers distributed a study invitation document to eligible participants. The document described the purpose of the study and included a QR code linking to an online questionnaire. The invitation document was prepared in six languages: Japanese, English, Chinese, Spanish, Portuguese, and Vietnamese. The online questionnaire allowed respondents to select their preferred language from these six options. An informed consent item was presented at the beginning of the online questionnaire. Participants were provided with an explanation of the study, and only those who indicated their consent electronically were allowed to proceed to the survey.

### 2.3. Survey Items

The survey collected information on the following variables: gender, age group, current nationality, current place of residence, length of residence in Japan, Japanese language proficiency, employment status, history of COVID-19 infection, COVID-19 vaccination status, use of government support and satisfaction with government support for foreign residents.

### 2.4. Analysis Method

Satisfaction with government support was measured using a 10-point scale. The median satisfaction score was calculated and found to be 7 points. Based on this value, satisfaction was dichotomized into two categories: high satisfaction (≥7 points) and low satisfaction (<7 points). Signal Detection Analysis (SDA) was conducted to identify the branching structure of explanatory variables that predicted levels of satisfaction. SDA is a decision-tree–based analytical method that clarifies conditional structures associated with outcome classification. In this study, branching conditions and classification accuracy were examined to explore the factor structure influencing satisfaction with government support. Furthermore, only fully completed questionnaires were included in the final dataset. For non-mandatory variables, missing data were handled by analyzing valid responses only, and cases with missing values were excluded from the specific computations. All statistical analyses were performed using IBM SPSS Statistics version 29.

### 2.5. Ethical Considerations

This study was approved by the Research Ethics Committee of Kyosei Studies, Graduate School of Human Sciences, The University of Osaka, on 17 September 2021 (Approval No. OUKS2124). All procedures were conducted in accordance with the ethical standards of the institutional research committee and the Declaration of Helsinki.

## 3. Results

### 3.1. Characteristics of Foreign Residents and COVID-19 Infection (Table 1)

This study analyzed the demographic characteristics of foreign residents. A total of 427 participants were included. Among them, 238 were female (55.7%) and 176 were male (41.2%). The largest age group was individuals in their 30s (46.4%), followed by those in their 40s (21.5%). The most common nationalities were the United States (15.3%), China (12.2%), and Brazil (11.3%). Most participants resided in Tokyo (38.6%), followed by Kanagawa (12.4%) and Chiba (8.7%). The most common length of residence was 10–19 years (30.2%), followed by 4–6 years (24.8%). Mean Japanese language proficiency scores were 6.4 for speaking, 5.5 for reading, 4.7 for writing, and 6.9 for listening. Full-time employment was the most common (57.3%). A total of 247 participants (57.8%) reported a history of COVID-19 infection.

**Table 1:** Characteristics of Foreign Residents (*n* = 427).

		*n* Mean	% *SD*
Sex/Gender	Female	238	55.7
	Male	176	41.2
	Other	1	0.2
	Prefer not to answer	12	2.8
Age group	20s	84	19.7
	30s	198	46.4
	40s	92	21.5
	50s	40	9.4
	60s	13	3.0
Nationality	United States	65	15.3
	China	52	12.2
	Brazil	48	11.3
	Vietnam	32	7.5
	UK	27	6.3
	Other	202	47.4
Current prefecture of residence	Tokyo	165	38.6
	Kanagawa	53	12.4
	Chiba	37	8.7
	Saitama	31	7.3
	Osaka	26	6.1
	Other	115	26.9
Length of residence in Japan	Less than 1 year	3	0.7
	1–3 years	43	10.1
	4–6 years	106	24.8
	7–9 years	87	20.4
	10–19 years	129	30.2
	20 years or more	59	13.8
Self-rated Japanese language proficiency ^†^	Speaking	6.4	2.3
	Reading	5.5	2.4
	Writing	4.7	2.5
	Listening	6.9	2.2
Current employment status	Full-time job	243	57.3
	Part-time job	52	12.3
	Freelance	42	9.9
	Student	42	9.9
	Not working	18	4.2
	Self-owned business	15	3.5
	Dependent	10	2.4
	Technical intern trainee	1	0.2
	Prefer not to answer	1	0.2
History of COVID-19 infection	Yes	247	57.8
	No	180	42.2

Note: Percentages were calculated based on valid responses only; missing values included non-response and not applicable. ^†^ 10 pts: As good as my first language, 0 pt: No skill at all.

### 3.2. COVID-19 Vaccination Status Among Foreign Residents (Table 2)

COVID-19 vaccination was reported by 400 participants (93.7%). Three times vaccine doses were received by 205 participants (51.2%), and more than three times doses by 112 participants (28.0%). The most common vaccination sites were municipal vaccination sites (137 participants, 37.6%) and workplaces (e.g., factories or companies) (120 participants, 33.0%). A total of 44 participants (11.0%) reported problems regarding COVID-19 vaccination. The most common concerns were concerns about side effects (9 participants, 33.3%), concerns about infection risk at the vaccination venue (5 participants, 18.5%), and delay in receiving the vaccination voucher (4 participants, 14.8%). Twenty-seven participants (6.3%) were unvaccinated. The most common reasons for non-vaccination were reluctance to receive the COVID-19 vaccination (5 participants, 38.5%) and concerns about side effects (4 participants, 30.8%).

**Table 2:** COVID-19 Vaccination Status among Foreign Residents (*n* = 427).

		*n*	%
Vaccination status	Yes	400	93.7
	No	27	6.3
Number of doses administered ^†^	1 time	3	0.8
	2 times	80	20.0
	3 times	205	51.2
	More than three times	112	28.0
Place of COVID-19 vaccination ^†^	Municipal mass vaccination site	137	37.6
	Workplace	120	33.0
	Primary care clinic	100	27.5
	Overseas	7	1.9
Problems regarding COVID-19 vaccination ^†^	Yes	44	11.0
	No	356	89.0
Types of problems regarding COVID-19 vaccination ^‡^	Concerns about side effects	9	33.3
	Concerns about infection risk at the vaccination venue	5	18.5
	Delay in receiving the vaccination voucher	4	14.8
	Uncertainty about where to get vaccinated	3	11.1
	Difficulty making a reservation	3	11.1
	Difficulty understanding explanations and instructions in Japanese	2	7.4
	Uncertainty about how to obtain the vaccination voucher	1	3.7
Reasons for not receiving vaccinated ^§^	Reluctance to receive COVID-19 vaccination	5	38.5
	Concerns about side effects	4	30.8
	Constitution/pregnancy	2	15.4
	Doubts about vaccine effectiveness	1	7.7
	Financial constraints	1	7.7

Note: Percentages were calculated based on valid responses only; missing values included non-response and not applicable. ^†^ Only participants who had been vaccinated against COVID-19. ^‡^ Only participants who experienced problems related to COVID-19 vaccination. ^§^ Only participants who had not been vaccinated against COVID-19.

### 3.3. Awareness, Utilization, and Satisfaction with Government Support (Non-Healthcare-Related)

#### 3.3.1. Awareness, Utilization, and Satisfaction with Government Support (Table 3)

A total of 213 participants (49.9%) reported being aware of and having used government support related to COVID-19. In contrast, 130 participants (30.4%) were aware of such government support but had not used it, and 84 participants (19.7%) were neither aware of nor had used these services. Among service users, the most common type of support received was financial assistance (115 participants, 79.9%), followed by material support (27 participants, 18.8%) and psychological support (2 participants, 1.4%). Among non-users, the most frequently cited reasons for not using government support were lack of necessity (59 participants, 56.7%), lack of knowledge regarding application procedures (19 participants, 18.3%), and the belief that they were not eligible for the services (17 participants, 16.3%). Satisfaction with government support was assessed using a 10-point scale. The mean satisfaction score was 5.8.

**Table 3:** Awareness, Utilization, and Satisfaction with Government Support (*n* = 427).

		*n* Mean	% *SD*
Use of government COVID-19 support measures	Used/currently using the measures	213	49.9
	Aware of the measures but not used	130	30.4
	Not aware of the measures	84	19.7
Types of government COVID-19 support used ^†^	Financial support	115	79.9
	Material support	27	18.8
	Mental health support	2	1.4
Reasons for not using government COVID-19 support ^‡^	Did not need the support	59	56.7
	Lack of knowledge about the application procedure	19	18.3
	Lack of awareness of eligibility	17	16.3
	Unsuccessful application	6	5.8
	Emotional reluctance to use the support	3	2.9
Satisfaction with government COVID-19 support measures ^§^		5.8	2.6

Note: Percentages were calculated based on valid responses only; missing values included non-response and not applicable. ^†^ Only participants who reported having received government COVID-19 support. ^‡^ Only participants who were aware of government COVID-19 support measures had not used them. ^§^ 10-point scale: Higher satisfaction yields a higher score.

#### 3.3.2. Determinants of Satisfaction with Government Support Identified by SDA (Figure 1)

Satisfaction scores were dichotomized using the median value of 7 points. Participants scoring 7 points or higher were classified into the high satisfaction group. Overall, 43.1% of participants belonged to the high satisfaction group. SDA identified awareness of government support as the primary splitting variable. Among participants who were aware of government support—regardless of actual use—the proportion of the high satisfaction group was 49.3%. In contrast, among participants who were neither aware of nor had used government support, the proportion of the high satisfaction group was 17.9%. The second splitting variable was the length of residence in Japan. Among participants with six years or less of residence, 61.7% belonged to the high satisfaction group. Among those with seven years or more of residence, 42.6% belonged to the high satisfaction group. Participants were classified into three groups: Group 1, those aware of government support services with six years or less of residence (*n* = 120); Group 2, those aware of government support with seven years or more of residence (*n* = 223); and Group 3, those not aware of government support services (*n* = 84).

**Figure 1:**
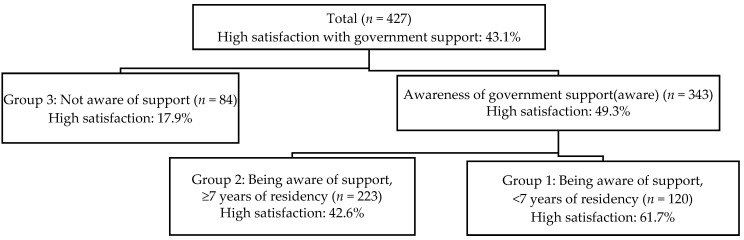
Determinants of Satisfaction with Government Support Identified by SDA (*n* = 427).

#### 3.3.3. Characteristics by Awareness of Government Support and Length of Residence (Table 4)

Participants in Group 1 were more likely to be in their 20s, whereas participants in Group 2 were predominantly in their 40s to 60s. Participants in Group 3 were most frequently in their 30s. Group 1 included a higher proportion of individuals from Southeast Asia and Africa. Group 2 included more individuals from North America and Latin America. Group 3 included more individuals from Western Europe. Japanese language proficiency was lower in Group 1 and higher in Group 2. Group 1 included more students and part-time workers, whereas Group 2 included more full-time employees and freelancers. Group 3 included more unemployed individuals and dependents.

**Table 4:** Characteristics by Awareness of Government Support and Length of Residence (*n* = 427).

		Group 1 (*n* = 120)		Group 2 (*n* = 223)		Group 3 (*n* = 84)		
		*n* Mean	% *SD*	*n* Mean	% *SD*	*n* Mean	% *SD*	*p*
Sex/gender ^†^	Female	68	59.1	123	56.4	47	58.0	0.888
	Male	47	40.9	95	43.6	34	42.0	
Age group ^‡^	20s	59	49.2	14	6.3	11	13.1	<0.001
	30s	49	40.8	107	48.0	42	50.0	
	40s	8	6.7	63	28.3	21	25.0	
	50s	2	1.7	30	13.5	8	9.5	
	60s	2	1.7	9	4.0	2	2.4	
Nationality ^†^	East Asia	21	17.6	40	18.0	21	25.3	0.308
	Southeast Asia	34	28.6	35	15.8	9	10.8	0.002
	South Asia	7	5.9	6	2.7	6	7.2	0.161
	Middle East and West Asia	0	0.0	1	0.5	1	1.2	0.469
	Western Europe	13	10.9	32	14.4	20	24.1	0.033
	Northern Europe	0	0.0	2	0.9	1	1.2	0.533
	Central and Eastern Europe	2	1.7	8	3.6	2	2.4	0.575
	North America	14	11.8	52	23.4	14	16.9	0.028
	Latin America	16	13.4	38	17.1	5	6.0	0.044
	Africa	9	7.6	4	1.8	2	2.4	0.019
	Oceania	3	2.5	4	1.8	2	2.4	0.890
Language proficiency ^‡^	Speaking	5.4	2.3	6.9	2.1	6.3	2.1	<0.001
	Reading	4.8	2.3	5.9	2.4	5.3	2.5	<0.001
	Writing	4.2	2.3	5.1	2.6	4.6	2.6	0.015
	Listening	5.8	2.3	7.4	2.0	7.1	2.1	<0.001
Employment status ^†^	Not working	3	2.5	8	3.6	7	8.3	0.106
	Full-time job	55	46.6	139	62.9	49	58.3	0.015
	Part-time job	17	14.4	23	10.4	12	14.3	0.466
	Freelance	1	0.8	32	14.5	9	10.7	<0.001
	Student	37	31.4	3	1.4	2	2.4	<0.001
	Self-owned business	0	0.0	13	5.9	2	2.4	0.017
	Technical intern trainee	1	0.8	0	0.0	0	0.0	0.274
	Dependent	4	3.4	3	1.4	3	3.6	0.361

Note: Percentages were calculated based on valid responses only; missing values included non-response and not applicable. ^†^ *χ*^2^ test ^‡^ One-way ANOVA.

## 4. Discussion

### 4.1. COVID-19 Vaccination Uptake and Access to Vaccination Support Among Foreign Residents

In this study, the COVID-19 vaccination rate among foreign residents was higher than the national average vaccination rate in Japan (approximately 80%) [8]. Although vaccination was not legally mandatory in Japan, practical circumstances—such as quarantine requirements upon re-entry into Japan for unvaccinated individuals—may have made vaccination effectively close to mandatory for many foreign residents. The survey results also suggest that administrative support related to vaccination was widely recognized. Only one respondent reported difficulty understanding how to receive the vaccination voucher, indicating that vaccination support was broadly disseminated among foreign residents. However, several barriers were still reported, including delays in receiving vaccination vouchers, uncertainty about where to get vaccinated, difficulty making reservations, and difficulty understanding explanations and instructions in Japanese. Because vaccination is a critical public health intervention during infectious disease outbreaks, it is essential to ensure that no residents—including foreign residents—are left behind. These findings suggest the need for improved multilingual communication and accessible reservation systems in preparation for future pandemics. Furthermore, it is important to highlight the critical role of the healthcare system in operationalizing governmental support. Our findings show that 27.5% of the respondents received their vaccinations at primary care clinics. This indicates that local medical institutions and public health centers function as the primary access points where governmental public health policies are delivered to foreign residents. The effective delivery of public support measures, such as the vaccination program, heavily relies on the robust collaboration between administrative bodies and the community healthcare system. Moreover, it is important to acknowledge that the reasons for non-vaccination among foreign residents often extend beyond logistical or language barriers. As highlighted by recent literature, religious, cultural, and moral values can significantly influence individuals’ vaccination choices and their perception of public health interventions [9]. In our study, a subset of unvaccinated respondents cited reluctance or doubts about vaccine effectiveness. These attitudes may be deeply intertwined with cultural or religious backgrounds that shape health behaviors. Recognizing these additional factors is crucial, as culturally and religiously sensitive communication, often mediated through community leaders, may be necessary to address vaccine hesitancy and improve the equitable reach of government support.

### 4.2. Pathways Through Which Awareness of Government Support Services and Length of Residence Shape Satisfaction

This study identified awareness of government support as a primary determinant of satisfaction. Higher awareness was associated with greater satisfaction regardless of whether participants used the services. Prior evidence suggests that access and availability are important factors influencing satisfaction with healthcare [10] and that perceived service quality is associated with healthcare utilization and may also influence satisfaction [11]. When individuals are aware of and use government support services, direct experiences of support may enhance their recognition of its contribution to daily life, thereby increasing satisfaction.

Previous research has demonstrated that the perceived availability of support plays a critical role in promoting psychological well-being and life satisfaction [12,13]. Building on this, it could be hypothesized that the mere awareness of available administrative support might function as a psychological reassurance or a perceived social safety net. Although trust and reassurance were not directly measured in our survey, recognizing that assistance is available if needed may provide a sense of security and control, fostering trust in local administrations. Our findings suggest that administrative support, like health insurance coverage, functions not only as an instrumental resource but also as a perceived safety net. This awareness may enhance a sense of security and reassurance, thereby increasing satisfaction even in the absence of direct service utilization—a mechanism also implied by recent literature [14].

These findings are consistent with previous studies on public responses during the COVID-19 pandemic, which suggest that satisfaction is influenced not only by policy effectiveness but also by perceptions of accessibility and government performance [15,16]. This implies that perceived availability and accessibility of support programs may enhance satisfaction by reinforcing the perception that authorities are responsive to citizens’ needs.

Awareness of government support services may also reflect exposure to government communication. Transparent communication and the provision of reliable information have been shown to strengthen trust in government during crises. Mansoor (2021) demonstrated that high-quality information provided through government communication channels contributes to strengthening citizens’ trust in government during the COVID-19 pandemic [17]. Thus, awareness may reflect not only the existence of support systems but also the effectiveness of communication, which may foster reassurance and institutional trust.

Furthermore, effective dissemination of information is essential. In addition to the existence of support systems, clear information regarding eligibility criteria and application procedures is necessary to ensure that support reaches those in need. Previous research has shown that awareness and utilization of services are influenced by individual and contextual factors, including access to information and personal conditions [18]. Without accessible and understandable information, even well-designed systems may fail to reach potential beneficiaries. Limited awareness may therefore act as a barrier to service utilization and influence individuals’ evaluations of support [19].

This study also showed that the length of residence influenced satisfaction. Differences in satisfaction may reflect changes in social integration, stability, and perceived need for support over time. Participants with <7 years of residence may still be establishing their living foundations, and government support may provide direct and immediate benefits, thereby increasing satisfaction. In contrast, those with ≥7 years of residence may experience more stable conditions and reduced dependence on support, leading to lower perceived benefits and satisfaction. Previous research has similarly reported that the determinants of residential satisfaction among foreign residents differ according to the length of residence and stage of settlement [20].

### 4.3. Characteristics of the Three Groups Determining Satisfaction (The <7 Years of Residency, ≥7 Years of Residency, and Those Unaware of Services)

This study identified distinct profiles across three groups defined by length of residence and awareness of government support. The group with <7 years of residence included a higher proportion of individuals in their 20s, as well as students and part-time workers, suggesting relatively unstable living foundations. This group also included more individuals from Southeast Asia and Africa, many of whom had limited Japanese language proficiency. Despite these vulnerabilities, it is plausible that individuals in this group may perceive a higher need for governmental support, which could hypothetically increase their motivation to seek information and engage with available services. Previous research suggests that immigrants’ subjective well-being is shaped by economic conditions, social networks, and information environments, all of which evolve over time [21]. In this context, individuals in earlier stages of settlement may be more actively engaged in seeking resources necessary for daily life. As a result, awareness of government support may be more easily developed, and once recognized, such services may function as concrete and immediate support, thereby increasing satisfaction.

The group with ≥7 years of residence included a higher proportion of individuals aged 40–60 years and those engaged in stable employment (e.g., full-time work and freelance work). This group also included relatively more individuals from North America and Latin America. These characteristics may reflect more stable living conditions and greater familiarity with Japanese society. Previous research has shown that limited social support and barriers to healthcare access are associated with poorer mental health and reduced well-being among immigrants in Japan [22]. Conversely, individuals with more stable socioeconomic conditions and established social networks may experience fewer immediate challenges in daily life. While such conditions may facilitate access to and understanding of government support information, the perceived need for support may be lower. As a result, although awareness may be maintained, the perceived benefits of support may be less salient, leading to relatively lower satisfaction compared with the <7 years of residence group.

The group that was unaware of government support included a higher proportion of individuals in their 30s. Individuals in this life stage may prioritize family responsibilities and childcare, which can constrain employment opportunities and increase the likelihood of being unemployed or categorized as dependents. This group also included more individuals from Western Europe. We hypothesize that differences in socioeconomic status, educational background, and social capital could influence patterns of information-seeking behavior and reliance on governmental support. Individuals with relatively higher levels of social capital might depend less on public support systems and therefore could be less attentive to available services. In addition, previous research suggests that awareness of services is influenced by individual and contextual factors, including access to information and personal circumstances [18]. A lack of awareness may limit opportunities to obtain reassurance from existing support systems and to benefit from available services, which may ultimately contribute to lower satisfaction.

### 4.4. Implications for Government Support Approaches by Awareness and Length of Residence

This study suggests that support strategies should differ according to awareness status and length of residence. The group with <7 years of residency who were aware of services included many individuals in their 20s, students and part-time workers, participants from Southeast Asia and Africa, and those with limited Japanese proficiency. For this group, multilingual information provision through immigrant community organizations and educational institutions may be effective. Practical formats include leaflets, videos, and audio materials. This is consistent with previous evidence suggesting that the use of patients’ native languages improves satisfaction, treatment adherence, and health outcomes [23]. In addition, training and deploying foreign peer supporters or navigators, as well as strengthening face-to-face assistance, may enhance awareness and translate service knowledge into tangible benefits in daily life. This aligns with previous evidence demonstrating that navigation interventions delivered by community health workers are effective in chronic disease management in primary care settings [24]. Together, these approaches may support immediate improvements in daily life and increase satisfaction.

For the group with ≥7 years of residence who were aware of services, many were aged 40–60 years, originated from North America or Latin America, had higher Japanese proficiency, and were engaged in stable employment (e.g., full-time work or freelancing). For this group, strengthening information dissemination through workplace networks and organizations may be effective. Additionally, providing detailed information about services, simplifying procedures, and improving usability may help maintain awareness while increasing the likelihood of utilization. Although this group may perceive less need for support, ensuring transparency and fairness in information provision may prevent declines in satisfaction and strengthen trust in government support systems. This is consistent with previous research indicating that culturally responsive care enhances trust in healthcare systems and improves patient satisfaction [25], and a similar principle may be applicable to government support delivery.

Several factors may contribute to limited awareness of government support among this group, including differences in educational background, personal social networks, and opportunities to encounter information in daily life. This is consistent with previous research showing that immigrants tend to rely on informal interpersonal information channels, such as family members, friends, and community organizations, when seeking information about services in host societies [26].

Studies have also highlighted the important role of community-based institutions as accessible information hubs for migrant populations. Local community centers, consultation services, and settlement support organizations serve as key access points through which migrants obtain information about public services and local resources [27]. These community-based venues allow individuals to encounter information naturally in the course of everyday life, which may reduce barriers to accessing administrative services.

From a policy perspective, strengthening community-based information channels may therefore be an effective strategy to improve awareness of government support services among foreign residents. Providing information through multicultural community hubs, consultation counters, and peer-support networks may help ensure that administrative support systems reach individuals who might otherwise remain unaware of available services.

For the group unaware of services, which included many individuals in their 30s, participants from Western Europe, and individuals who were unemployed or dependents, face-to-face informational sessions in everyday community settings may be particularly important. Potential venues include multicultural community hubs and local salons, where individuals can encounter information as part of their daily routines. Given the lower reliance on government support in this group, framing information as a source of reassurance and an opportunity for social participation may increase awareness and prevent further declines in satisfaction. This is consistent with a systematic review demonstrating that community navigators are effective in improving health outcomes among immigrants [28]. Disseminating information via peer supporters and community networks may reduce psychological distance from administrative systems and encourage willingness to use available services. In addition to administrative efforts, the healthcare system plays an indispensable role as an information hub. Healthcare professionals, including doctors, nurses, and community health workers in primary care settings, are often the most trusted and accessible point of contact for foreign residents. Therefore, the healthcare system serves not merely as a provider of medical treatment but as a critical bridge connecting vulnerable populations to broader governmental support. As previous evidence suggests, integrating culturally responsive care and utilizing community health workers as navigators within the healthcare system not only improves clinical health outcomes but also strengthens trust in public institutions. Enhancing the synergy between government policies and the healthcare delivery system is essential for maximizing the reach and satisfaction of public support for foreign residents [24,27,28].

Beyond the domestic context in Japan, our findings resonate with international evidence regarding the psychological and socioeconomic vulnerabilities of migrant populations and international students during the COVID-19 pandemic. Research across multiple countries has highlighted that foreign populations experienced significantly elevated stress and anxiety levels. For instance, a cross-sectional study of international medical students in Serbia found that a majority experienced higher-than-usual stress levels, primarily driven by concerns over their finances, academic future, and the health of loved ones in their home countries [29]. Similarly, global scoping reviews and studies from host countries such as Canada have demonstrated that migrant workers and international students faced disproportionate mental health burdens due to social isolation, job insecurity, and exclusion from national emergency financial relief programs [30,31]. Furthermore, structural barriers and the lack of culturally tailored support often exacerbated their difficulties in accessing crucial public health information and administrative services [32]. To cope with these unprecedented challenges, accessible institutional support, transparent communication, and community networks emerged as critical protective factors that mitigate psychological distress. Comparing our local findings with these international results underscores a universal trend: awareness of the availability of support provides a vital psychological safety net. Ensuring equitable access to information and structural support is therefore not only a localized issue in Japan but a shared global challenge essential for safeguarding the well-being of foreign residents during public health crises.

Several limitations should be considered when interpreting these findings. First, participants were recruited through a commercial online panel managed by an online survey company. Web-based surveys and opt-in online panels are subject to potential coverage errors and self-selection bias, which can limit the representativeness of the sample and the generalizability of estimates to the broader foreign resident population. In addition, participation required internet access and sufficient digital literacy, which may have resulted in the underrepresentation of individuals with limited internet access or those facing greater time and resource constraints, potentially introducing selection bias [33]. Second, the recruitment approach may have disproportionately captured foreign residents with relatively stable living conditions (e.g., registered residents with more stable residence situations). Consequently, foreign residents in more vulnerable circumstances—such as those with unstable residence status—may not have been adequately represented. Prior research in Japan indicates that some migrant groups experience barriers to healthcare access, including gaps in insurance coverage and other structural barriers, suggesting that determinants of awareness, utilization, and satisfaction may differ among harder-to-reach and more vulnerable populations who are often underrepresented in survey-based studies [34,35,36]. Future studies should consider complementary recruitment strategies (e.g., community organizations, multilingual outreach, and mixed-mode surveys) to better include these populations and to evaluate whether the observed associations hold across varying levels of vulnerability.

## 5. Conclusions

This study demonstrated that awareness of government support was strongly associated with higher satisfaction in this sample. Individuals who used government support reported higher satisfaction, likely through direct support effects. Even among those who did not use the services, satisfaction was higher, which may reflect reassurance derived from the existence of available systems and trust in government support institutions. In contrast, individuals who were unaware of government support tended to report lower levels of satisfaction.

The study also showed that the length of residence was an additional factor associated with satisfaction with government support, potentially by shaping perceived need and perceived benefits. The <7-year residence group tended to have less stable living foundations and experienced stronger perceived benefits from government support, which might have contributed to higher satisfaction. Conversely, the ≥7 years of residence group generally had more stable living conditions and showed reduced dependence on government support, which may relate to relatively lower satisfaction.

Furthermore, this study identified distinct characteristics among the <7 years residence group, the ≥7 years residence group, and individuals who were unaware of government support. These groups differed in age distribution, region of origin, Japanese language proficiency, and employment status. Based on these findings, the study suggests that tailored policy measures are required for each group. Such measures include multilingual support, the deployment of foreign peer supporters, simplification of government support procedures, and information dissemination through community-based interaction hubs. Implementing these strategies in a stratified manner according to population characteristics is essential to improve satisfaction with government support.

As post-pandemic international mobility resumes, the number of foreign residents in Japan has rapidly rebounded, reaching approximately 3.76 million by the end of December 2024. This population reflects increasing diversity in nationalities and residence statuses, with significant concentrations in major metropolitan areas where employment opportunities are abundant [1]. At a time when Japan is introducing new policies to reshape its foreign labor system, including the planned transition from the Technical Intern Training Program to the Developmental Employment system, it is particularly important to reflect on the experiences of foreign residents during the COVID-19 pandemic [37]. The lessons learned from administrative support during this period should be carefully examined and incorporated into future policy development to build more inclusive and responsive support systems for this growing and diverse population.

## Data Availability

The data presented in this study are not publicly available due to ethical restrictions and the protection of participants’ privacy.
